# Excess Thermodynamic Properties and FTIR Studies of Binary Mixtures of Toluene with 2-Propanol or 2-Methyl-1-Propanol

**DOI:** 10.3390/molecules29194706

**Published:** 2024-10-04

**Authors:** Maria Magdalena Naum, Vasile Dumitrescu

**Affiliations:** Chemistry Department, Petroleum and Gas University of Ploiesti, 100680 Ploiesti, Romania; vdumi@upg-ploiesti.ro

**Keywords:** density, viscosity, excess properties, PFP theory, FTIR spectra

## Abstract

Physical properties of the binary solutions, toluene with 2-propanol and 2-methyl-1-propanol, were measured at T = 293.15, 298.15, 303.15, 308.15, and 313.15 K and P = 100 kPa. The experimental density values were tested with the Emmerling et al. and Gonzalez-Olmos–Iglesias equations. The results indicate that the equation by Emmerling et al. is the best to correlate the density for toluene + 2-methyl-1-propanol system, while for toluene + 2-propanol, both proposed equations are proper to correlate the density with composition and temperature. The viscosity results were verified with different models containing two adjustable parameters. The values of viscosity deviation (∆η), excess molar volume (*V*^E^), excess Gibbs energy (ΔG^*E^), partial molar volumes ($\bar{V_{1}}$ and $\bar{V_{2}}$), and apparent molar volume ($V_{\varphi ,1}$ and $V_{\varphi ,2}$) were calculated. The values of the excess molar volume were positive for both systems, while negative values were obtained for the viscosity deviation and the excess Gibbs energy. The excess properties of the mixtures were adjusted to the Redlich–Kister equation. The values of thermodynamic functions of activation of viscous flow were computed and analyzed. Additionally, the Prigogine–Flory–Patterson (PFP) theory was applied to calculate *V*^E^ and then compared with experimental values. The values of the percentage absolute average deviation obtained suggest the validity of this theory. The Fourier transform infrared spectroscopy (FTIR) spectra of the binary solutions studied in this work allowed for the understanding of the interactions between the molecules of these systems.

## 1. Introduction

The rise in global temperatures and the diminishing supply of fossil fuels have prompted researchers to focus on alternative biofuels such as biodiesel, bioalcohol, vegetable oil, and other biomass sources [1,2].

Biofuels are utilized as on oxygenated substance in vehicle fuel. Fuel additives like alcohols, ethers, and esters that contain oxygenated compounds are utilized. The oxygen found in these substances aids in the full burning of the fuel, and these substances are also used as effective antiknocking agents with a high octane rating [3].

The addition of fuel oxygenates to gasoline, including those of biological origin, increases the combustion temperatures and improves engine efficiency, and thus the levels of carbon monoxide and unburned hydrocarbons decrease in the auto exhaust [4].

Gasoline blend components derived from C3–C5 alcohols made from renewable sources show promise as biofuel additives generated through the microbial fermentation of biomass [5]. *n*-Butanol and *i*-butanol (2-methyl-1-propanol) are the preferred options for butanol production from agricultural feedstocks. While *n*-butanol has been extensively researched as a motor fuel, there is increasing interest in the production of *i*-butanol from biomass sources for use as a fuel [6,7].

Aromatic hydrocarbons are also found in gasoline. Nevertheless, because benzene is carcinogenic, it must be substituted; toluene is a suitable alternative since it shares similar properties with benzene but is less carcinogenic [8]. The use of alcohol and toluene mixtures in fuel formulation improves air quality, increases their efficiency, and improves the octane/cetane number.

The polar head of mono alcohol shows a preference for interacting with the delocalized *π*-electrons of toluene. The characteristics such as density, transport, surface tension, etc., of these liquid solutions are highly important in the chemical design and formulation of oxygenated fuel [9]. Studying the molecular interactions and arrangements in liquid mixtures is crucial due to the significant impact of thermophysical and thermodynamic properties on deviations from ideality [10].

Various types of intermolecular interactions such as polar, non-polar, ionic, and possible combinations of these interactions can affect the behavior of liquid solutions. Regarding this matter, excess molar properties are a useful topic for gathering data in preparation for modeling solution properties [11].

In this paper, the densities and viscosities of toluene + 2-propanol or 2-methyl-1-propanol mixtures were measured at T = 293.15–313.15 K and P = 100 kPa over the entire concentration range. Some properties of these systems were also determined by other researchers but not in the same conditions. Mahendran et al. [12] studied the ultrasonic velocity, density, and viscosity at 303 K for the ternary system of aniline + toluene + iso-butanol and the binary systems, while Verma et al. [9] investigated the refractive index and speed of sound for the binary mixture of an isomer of butanol with cyclohexane, benzene, and toluene at 308.15 K. Gahlyan et al. [13] reported the viscosities of 2-propanol + hydrocarbons at 298.15 K and 308.15 K, and Verma et al. [14] reported the values of viscosities for butanol isomers with cyclohexane or benzene or toluene at 308.15 K.

Ramana et al. [15] studied the dielectric and excess dielectric constants of toluene with alcohols at 303, 313, and 323 K, and Bhardwaj et al. [16] reported the excess molar volumes of benzene or toluene with an isomer of butanol at the temperature of 308.15 K. Swamy et al. [17] investigated the excess volumes of toluene with 1-propanol, 2-propanol, 1-butanol, 2-methyl-1-propanol, 1-pentanol, 1-hexanol, 1-heptanol, and cyclohexanol at 303.15 K, while Yadav et al. [18] reported the excess volumes of propanol or 1-methylethanol + benzene or toluene or o-, m-, or p-xylene at the temperature of 308.15 K.

The excess properties (*V*^E^, ΔG^*E^ and ∆η) were computed and subsequently correlated with the Redlich–Kister equation. The *V*^E^ were correlated using the Prigogine–Flory–Patterson (PFP) model. The thermal expansion coefficient and activation thermodynamic functions of viscous flow were computed and examined. FTIR analysis of binary mixtures of toluene or *n*-heptane with 2-propanol and 2-methyl-1-propanol was conducted to investigate the interactions among the molecules.

## 2. Results and Discussion

### 2.1. Density and Viscosity

Our results of densities and viscosities for pure toluene, 2-propanol, and 2-methyl-1-propanol at T = 293.15–313.15 K are in good concordance with the values from the literature and are presented comparatively in Table 1.

Density values of toluene from our experiments show differences of up to 0.15% compared to those found in sources [12,13,19,20,21,22], while viscosity values differ by a maximum of 2.2% when compared to the literature sources [12,14,19,20,22]. The density values for 2-propanol differ by up to 0.2% in various studies [11,13,23,24,25] and for 2-methyl-1-propanol, the difference is less than 0.4% according to the different sources [11,14,26,27,28,29].

Viscosity values found in other sources differ from the experimental data by a maximum of 2.1% for 2-propanol [11,13,23,24,25] and a maximum of 3% for 2-methyl-1-propanol [11,14,27,28,29]. The variations are due to the varying purity levels of the reagents utilized. Table 2 and Table 3 provide the experimental densities and viscosities for the mixtures, while the corresponding graphs are shown in Figures S1–S4.

The density values of the mixtures of toluene and 2-methyl-1-propanol were greater than those of toluene with 2-propanol mixtures at the identical temperature and concentration. The density values of 2-methyl-1-propanol were higher than those of 2-propanol because primary alcohols have a higher association of hydrogen bonds compared to secondary alcohols [24]. The density of mixtures decreased as the concentration of alcohols increased and decreased as the temperature increased at a constant concentration. As the amount of alcohol in the binary systems increased at a constant temperature, the viscosity also increased, whereas the viscosity of solutions decreased as the temperature increased at a constant concentration. As the temperature rose, the molecules became less cohesive, causing an acceleration in the molecular exchange rates and a decrease in viscosity [30].

**Table 1:** Comparison of experimental results of density (*ρ*) and viscosity (*η*) of the pure compounds with the literature values at T = 293.15–313.15 K.

Component	T/(K)	*ρ*/(g·cm^−3^)		*η*/(mPas)	
		This Work	Lit. Value	This Work	Lit. Value
Toluene	293.15	0.8665	0.86686 [19]	0.596	0.598 [19]
			0.866859 [20]		0.59610 [20]
	298.15	0.8622	0.86220 [19]	0.567	0.569 [19]
			0.862214 [21]		0.56703 [20]
			0.862206 [20]		
			0.86121 [13]		
	303.15	0.8579	0.85754 [19]	0.522	0.522 [22]
			0.857552 [21]		0.526 [12]
			0.857541 [20]		
			0.85755 [22]		
			0.8578 [12]		
	308.15	0.8537	0.85285 [19]	0.498	0.509 [19]
			0.852858 [21]		0.493 [22]
			0.852861 [20]		0.4980 [14]
			0.85384 [13]		
			0.85272 [22]		
	313.15	0.8493	0.84816 [19]	0.483	0.485 [19]
			0.84836 [21]		0.48657 [20]
			0.848164 [20]		
			0.84802 [22]		
2-propanol	293.15	0.7860	0.78525 [23]	2.414	2.3621 [23]
			0.78544 [11]		2.3621 [11]
			0.78535 [24]		2.414 [24]
	298.15	0.7825	0.78116 [13]	2.045	2.035 [13]
			0.78110 [24]		2.070 [24]
			0.7810 [25]		2.08 [25]
	303.15	0.7774	0.7768 [23]	1.791	1.8014 [23]
			0.77675 [11]		1.7694 [11]
			0.77712 [24]		1.785 [24]
			0.7767 [25]		1.79 [25]
	308.15	0.7730	0.77131 [13]	1.550	1.535 [13]
			0.77288 [24]		1.546 [24]
			0.7721 [25]		1.58 [25]
	313.15	0.7689	0.7680 [23]	1.327	1.3311 [23]
			0.76798 [11]		1.3297 [11]
			0.76879 [24]		1.347 [24]
			0.7678 [25]		1.34 [25]
2-methyl-1-propanol	293.15	0.8014	0.80175 [11]	3.929	3.943 [28]
			0.80148 [26]		4.0516 [27]
			0.80203 [27]		
			0.80168 [28]		
	298.15	0.7974	0.79816 [27]	3.408	3.4307 [27]
			0.798033 [29]		3.4023 [29]
			0.79774 [28]		3.332 [28]
	303.15	0.7920	0.79399 [11]	2.923	2.877 [11]
			0.79360 [26]		2.9226 [27]
			0.79425 [27]		2.884 [28]
			0.79432 [28]		
	308.15	0.7879	0.79056 [14]	2.554	2.5057 [14]
			0.78966 [26]		2.5053 [27]
			0.79031 [27]		
	313.15	0.7832	0.78604 [11]	2.187	2.091 [11]
			0.78572 [26]		2.1612 [27]
			0.78631 [27]		

Standard uncertainties: *u*(*p*) = 2 kPa, Expanded uncertainties: *U*(*η*) = 0.02 mPa·s and *U*(*ρ*) = 0.0006 g·cm^−3^ (0.95 of confidence).

**Table 2:** Density data *ρ*/(g cm^−3^) as a functions of mole fraction at T = 293.15–313.15 K and P = 100 kPa.

*x*_1_	*T*/(K)				
	293.15	298.15	303.15	308.15	313.15
toluene (1) + 2-propanol (2)					
0.1020	0.7949	0.7915	0.7866	0.7825	0.7785
0.2001	0.8031	0.7995	0.7949	0.7908	0.7869
0.3004	0.8110	0.8074	0.8029	0.7988	0.7949
0.4030	0.8188	0.8150	0.8107	0.8066	0.8026
0.5051	0.8263	0.8225	0.8182	0.8141	0.8102
0.6029	0.8338	0.8299	0.8257	0.8216	0.8176
0.7052	0.8422	0.8384	0.8342	0.8301	0.8262
0.7998	0.8503	0.8463	0.8421	0.8380	0.8339
0.9005	0.8585	0.8543	0.8501	0.8460	0.8417
toluene (1) + 2-methyl-1-propanol (2)					
0.1000	0.8072	0.8033	0.7981	0.7942	0.7896
0.1969	0.8129	0.8091	0.8041	0.8002	0.7957
0.3008	0.8192	0.8153	0.8105	0.8065	0.8021
0.3922	0.8248	0.8209	0.8161	0.8121	0.8077
0.4947	0.8313	0.8273	0.8226	0.8186	0.8142
0.5952	0.8377	0.8337	0.8291	0.8251	0.8207
0.6936	0.8443	0.8403	0.8358	0.8318	0.8275
0.7941	0.8514	0.8473	0.8429	0.8388	0.8345
0.8965	0.8587	0.8545	0.8502	0.8461	0.8417

Standard uncertainties: *u*(*x*_1_) = 3 × 10^−4^, *u*(*p*) = 2 kPa, *u*(*T*) = 0.05 K; Expanded uncertainties: *U*(*ρ*) = 0.0006 g·cm^−3^ (0.95 of confidence).

**Table 3:** Viscosity data *η*/(mPa s) as a functions of mole fraction at T = 293.15–313.15 K and P = 100 kPa.

*x*_1_	T/(K)				
	293.15	298.15	303.15	308.15	313.15
toluene (1) + 2-propanol (2)					
0.1020	1.720	1.468	1.294	1.171	1.013
0.2001	1.338	1.144	1.039	0.939	0.824
0.3004	1.072	0.942	0.865	0.797	0.714
0.4030	0.882	0.788	0.732	0.688	0.624
0.5051	0.770	0.697	0.653	0.609	0.570
0.6029	0.698	0.637	0.601	0.557	0.534
0.7052	0.647	0.594	0.566	0.530	0.507
0.7998	0.619	0.582	0.546	0.518	0.495
0.9005	0.608	0.575	0.543	0.506	0.487
toluene (1) + 2-methyl-1-propanol (2)					
0.1000	2.749	2.392	2.066	1.861	1.632
0.1969	2.132	1.848	1.636	1.476	1.297
0.3008	1.626	1.411	1.297	1.186	1.024
0.3922	1.316	1.146	1.074	0.992	0.862
0.4947	1.086	0.969	0.878	0.816	0.739
0.5952	0.923	0.869	0.765	0.708	0.667
0.6936	0.813	0.771	0.704	0.655	0.615
0.7941	0.717	0.689	0.635	0.594	0.558
0.8965	0.651	0.631	0.585	0.561	0.533

Standard uncertainties: *u*(*x*_1_) = 3 × 10^−4^, *u*(*p*) = 2 kPa, *u*(*T*) = 0.05 K; Expanded uncertainties: *U*(*η*) = 0.02 mPa·s (0.95 of confidence).

The densities of binary mixtures were described by the Emmerling et al. [31] (Equation (1)) and Gonzalez-Olmos–Iglesias [32] (Equation (2)) equations as follows:

(1) $$\rho =x_{1}\rho_{1}+x_{2}\rho_{2}+x_{1}x_{2}\left[\begin{array}{c} P_{1}+P_{2}T+P_{3}T^{2}+\left(P_{4}+P_{5}T+P_{6}T^{2}\right)\left(x_{1}-x_{2}\right)+\\ \left(P_{7}+P_{8}T+P_{9}T^{2}\right){\left(x_{1}-x_{2}\right)}^{2} \end{array}\right]$$

(2) $$\rho =\sum_{i=0}^{2}A_{i}x^{i}$$

The densities (*ρ*_i_) of each individual substance *i* in the Equation (1) vary with temperature according to the following equation:

(3) $$\rho_{i}=A_{i}+B_{i}T+C_{i}T^{2}\;\;\left(i=1,\;2\right)$$

In Equation (2), *A*_i_ is a polynomial temperature dependence function as follows:

(4) $$A_{i}=\sum_{j=0}^{2}A_{ij}T^{i}$$

The experimental data were used to estimate the adjustable parameters (*P*_1_–*P*_9_, *A_i_*, *B_i_*, *C_i_* and *A_ij_*) of the equations with the Levenberg–Marquardt algorithm [33].

Table S1 show the adjustable parameters and the standard deviation that was calculated using the following equation:

(5) $$\sigma ={\left[\frac{\sum {\left(X_{exp}-X_{calc}\right)}^{2}}{m-n}\right]}^{1/2}$$

where *X* is the value of the determined property, *m* is the number of experimental values, and *n* is the number of adjustable parameters.

The data indicate that the equation developed by Emmerling et al. is the most effective in relating the density of mixtures of toluene + 2-methyl-1-propanol to their composition and temperature and, for the toluene + 2-propanol system, both the Emmerling et al. and the Gonzales-Olmos–Iglesias equations correlate well with the density of the mixtures.

The density data were correlated with the polynomial equation as a function of temperature as in the following equation:

(6) $$\rho =a+bT+cT^{2}$$

where *ρ* and *T* are the density and temperature of the solution, respectively, and *a*, *b*, and *c* are the empirical parameters.

The isobaric thermal expansion coefficient ($\alpha_{p}$) gives valuable information regarding how the density changes in response to temperature increases at a constant pressure, as well as how much a component expands with temperature [34].

The isobaric thermal expansion coefficient is calculated with the following equation:

(7) $$\alpha_{p}=-\frac{1}{\rho }{\left(\frac{\partial \rho }{\partial T}\right)}_{p}=-\frac{1}{\rho }(2cT+b)$$

The data in Table S2 reveal that the $\alpha_{p}$ values rise as the temperature increases. The isobaric thermal expansion coefficient is influenced by the concentration of the solution components and is connected to how the volume of a solution changes with temperature. During heat transfer, the energy of intermolecular bonds between atoms fluctuates, and as temperature rises, the atoms’ thermal vibrations intensify [31]. As the toluene concentration increases, the $\alpha_{p}$ values decrease due to alcohol having a stronger molecular force than toluene.

The viscosity dependence on the concentration of components in binary solutions was represented using the two-parameter Wilson [35] (Equation (8)), Noda and Ishida [36] (Equation (9)), Eyring–NRTL [37] (Equation (10)), Eyring–Van Laar (Equation (11)) and Eyring–Margules (Equation (12)) [38] models.

(8) $$\ln\left(\eta V\right)=x_{1}\ln\left(\eta_{1}V_{1}\right)+x_{2}\ln\left(\eta_{2}V_{2}\right)+x_{1}\ln\left(x_{1}+x_{2}\frac{V_{2}}{V_{1}}\exp\left(-\frac{\lambda_{12}}{RT}\right)\right)+x_{2}\ln\left(x_{2}+x_{1}\frac{V_{1}}{V_{2}}\exp\left(-\frac{\lambda_{21}}{RT}\right)\right)$$

(9) $$\ln\left(\eta V\right)=x_{1}\ln\left(\eta_{1}V_{1}\right)+x_{2}\ln\left(\eta_{2}V_{2}\right)+x_{1}x_{2}\left[\frac{w_{12}}{x_{2}+x_{1}exp(\frac{-w_{12}}{RT})}+\frac{w_{21}}{x_{1}+x_{2}exp(\frac{-w_{21}}{RT})}\right]$$

(10) $$\ln\left(\eta V\right)=x_{1}\ln\left(\eta_{1}V_{1}\right)+x_{2}\ln\left(\eta_{2}V_{2}\right)+x_{1}x_{2}\left[\frac{\tau_{21}exp(-\alpha \tau_{21})}{x_{1}+x_{2}exp(-\alpha \tau_{21})}+\frac{\tau_{12}exp(-\alpha \tau_{12})}{x_{2}+x_{1}exp(-\alpha \tau_{12})}\right]$$

(11) $$\ln\left(\eta V\right)=x_{1}\ln\left(\eta_{1}V_{1}\right)+x_{2}\ln\left(\eta_{2}V_{2}\right)+\frac{Ax_{1}x_{2}}{x_{1}+Bx_{2}}$$

(12) $$\ln\left(\eta V\right)=x_{1}\ln\left(\eta_{1}V_{1}\right)+x_{2}\ln\left(\eta_{2}V_{2}\right)+x_{1}x_{2}\left(A_{21}x_{1}+A_{12}x_{2}\right)$$

where *η*, *η*_1_, and *η*_2_ represent the dynamic viscosities of the solutions and the individual components *x*_1_ and *x*_2_ are the mole fractions, *M*_1_ and *M*_2_ indicate the molecular masses, *V*, *V*_1_, and *V*_2_ are the molar volume of the solutions and of the pure components, *T* is the temperature, *R* is the gas constant; *λ*_12_, *λ*_21_, *w*_12_, *w*_21_, *τ*_12_,*τ*_12_, *A*, *B*, *A*_21_, and *A*_12_ are adjustable parameters. The Eyring–NRTL equation consists of three parameters, one of which is *α*, set as a value of 0.30 in this case.

The parameters were estimated using the Levenberg–Marquardt algorithm [33] and the mean absolute deviation (ADD%) between the experimental and calculated values was determined using the following equation:

(13) $$ADD\%=\frac{100}{m}\sum_{i=1}^{m}{\left|\frac{X_{exp}-X_{cal}}{X_{exp}}\right|}_{i}$$

where *X* is the value of the determined property and *m* is the number of experimental values.

The data presented in Table S3 show that for the toluene + 2-propanol system the maximum values for ADD are 0.7% for the Wilson equation and, respectively, 0.59% for the Noda and Ishida, while for the toluene + 2-methyl-1-propanol system, the maximum values for ADD are 1.55% for the Wilson equation and, respectively, 1.37% for the Noda and Ishida equation. By applying the Eyring–NRTL and Eyring–Van Laar equations, the maximum ADD values of 0.64% were obtained for the toluene + 2-propanol system and, respectively, 1.48% for the toluene and 2-methyl-1-propanol system. The Eyring–Margules equation gives the most accurate results for correlating the viscosity of the mixtures with their concentration, having the maximum ADD values of 0.57% for the toluene + 2-propanol system and 1.34%, respectively, for the toluene + 2-methyl-1-propanol system.

### 2.2. Thermodynamic Functions of Activation

The energies of activation of viscous flow were calculated with the following equations [39]:

(14) $$\eta =\frac{hN}{V}exp\left(\frac{∆G^{\neq }}{RT}\right)$$

(15) $$∆G^{\neq }=∆H^{\neq }-T∆S^{\neq }$$

where *η* is the viscosity of a mixture, *h* is Planck’s constant, *N* is Avogadro’s number, *V* is the molar volume of the mixture, *R* is the universal gas constant, *T* is temperature, $∆G^{\neq }$, $∆H^{\neq }$, and $∆S^{\neq }$ denote the molar Gibbs energy, enthalpy, and entropy of activation. In the temperature range of 293.15 to 313.15 K, the plots of ln(*ηV*/*hN*) against *1*/*T* show a linear relationship, allowing for the determination of enthalpy ($∆H^{\neq }$) and entropy ($∆S^{\neq }$) of the viscous flow from the slopes and intercepts. The Gibbs activation energy values ($∆G^{\neq }$) were also determined and shown in Figures S5 and S6. Table 4 lists the thermodynamic activation functions values and as can be seen, the $∆G^{\neq }$ and $∆H^{\neq }$ values are both positive for binary systems and decrease as the toluene concentration in the solution increases at a fixed temperature. The $∆G^{\neq }$ values reduce as the temperature rises for the alcohol-rich solutions, and they increase for the toluene-rich solutions with a high concentration. The values of $∆H^{\neq }$ for alcohols are nearly three times greater than for toluene, showing that $∆H^{\neq }$ increases by the association and dipole–dipole interactions. The difference in entropy is positive for alcohols and alcohol-rich solutions and negative for toluene and toluene-rich solutions. The breaking of the hydrogen bonds formed in alcohols lead to a structural disorder and positive values of $∆S^{\neq }$ [40].

### 2.3. Excess Properties

The experimental densities data were used to calculate the excess molar volumes through the following equation:

(16) $$V^{E}=\frac{\left[x_{1}M_{1}+x_{2}M_{2}\right]}{\rho }-\left[\frac{x_{1}M_{1}}{\rho_{1}}+\frac{x_{2}M_{2}}{\rho_{2}}\right]$$

The viscosity deviation values (Δ*η*) were determined by applying the following equation to the viscosity experimental data:

(17) $$\Delta \eta =\eta -(x_{1}\eta_{1}+x_{2}\eta_{2})$$

The excess Gibbs energy ($∆G^{\neq E}$) was determined using the following equation:

(18) $$∆G^{\neq E}=RT\left[ln\eta V-\left(x_{1}ln\eta_{1}V_{1}+x_{2}ln\eta_{2}V_{2}\right)\right]$$

The following Redlich–Kister equation [41] can be used to represent the excess properties in binary systems.

(19) $$X^{E}=x_{1}x_{2}\sum_{k=0}^{3}a_{k}{\left(2x_{1}-1\right)}^{k}$$

*X*^E^ can stand for *V*^E^, Δ*η*, or $∆G^{\neq E}$, while *a*_k_ signifies the polynomial coefficients which were calculated with the Levenberg–Marquardt algorithm [33].

**Figure 1:**
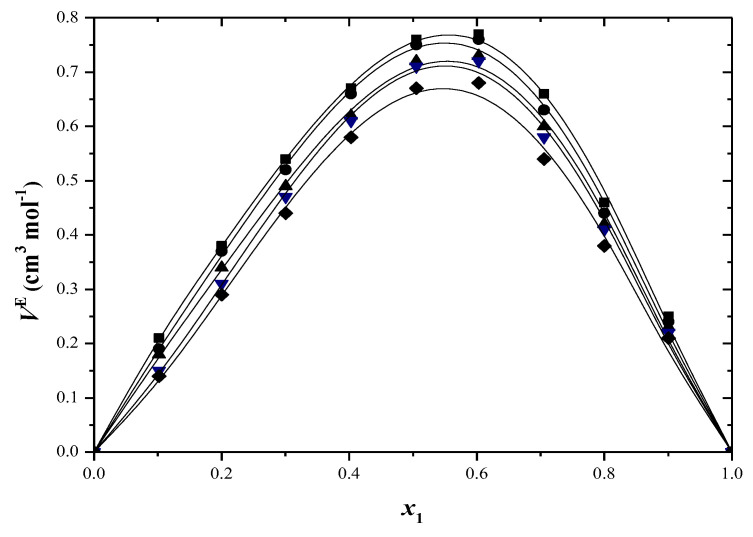
Excess molar volumes (*V*^E^) versus mole fraction for toluene (1) + 2−propanol (2) system at: ■ 293.15 K; ● 298.15 K; ▲ 303.15 K; ▼ 308.15 K; ♦ 313.15 K. The solid curve was determined with R–K equation.

**Figure 2:**
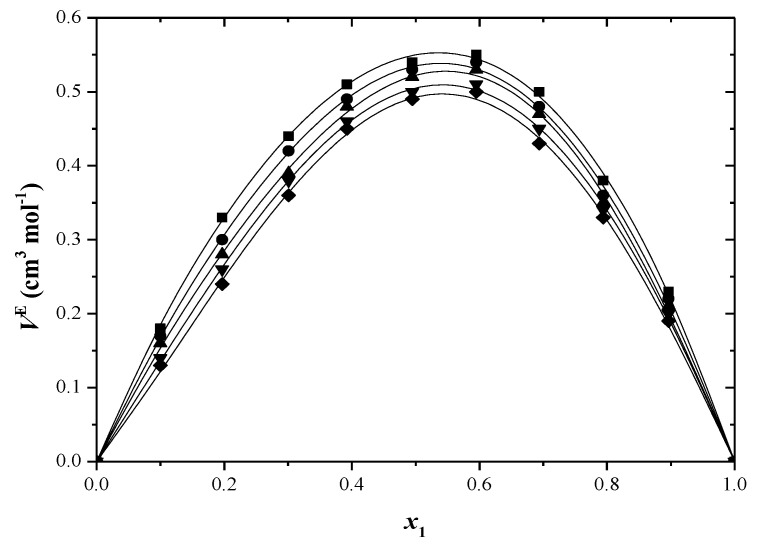
Excess molar volumes (*V*^E^) versus mole fraction for toluene (1) + 2−methyl−1−propanol (2) system at: ■ 293.15 K; ● 298.15 K; ▲ 303.15 K; ▼ 308.15 K; ♦ 313.15 K. The solid curve was determined with R–K equation.

Table S4 presents the excess molar volume results, and these are also shown in Figure 1 and Figure 2. At all temperatures, the excess molar volumes are positive for every binary system across the entire composition range.

Many factors, such as disturbances in liquid structure, negative group interactions, differences in molecule sizes and varying free volumes in liquids, can affect *V*^E^ values [42]. Positive molar volumes are obtained when the first two factors are present. The positive *V*^E^ values in toluene–alcohol systems indicate the absence of powerful intermolecular interactions. The rise in solution volume from mixing occurs due to the breaking of hydrogen bonds and dispersive interactions among various molecules. The introduction of a non-polar solvent disrupts hydrogen bonding in alcohol clusters, causing an increase in excess volume as the aggregates have a larger volume than their separate parts [43].

Toluene exhibits a low dipole moment due to the presence of the electron-donating methyl group in the aromatic ring. This created polarity serves as a barrier that prevents additional interactions, which may explain the positive *V*^E^ values [44]. The excess molar volumes decrease as the alcohol’s alkyl chain length increases. Toluene + 2-propanol mixtures have larger excess molar volumes than toluene mixed with 2-methyl-1-propanol. The impact of disrupting hydrogen bonds by introducing a non-polar solvent is more significant for 2-propanol than for 2-methyl-1-propanol. This mainly clarifies why the *V*^E^ are higher in solutions of 2-propanol than in solutions of 2-methyl-1-propanol. Ortega et al. [45] discovered that *V*^E^ values increase when alkanes are combined with secondary or tertiary alcohols in comparison to primary alcohols, and the experimental findings support this. The excess values decrease in positivity as the temperature rises. This could be because the interactions between similar molecules decrease more with temperature than the interactions between unlike molecules do [46].

The Δ*η* values can be clarified by taking into account the following two crucial factors: variations in the dimensions and forms of molecule parts and characteristic interactions among different molecules, like the formation of H-bonds and change transfer complexes and result in a greater increase in viscosity in solution, compared to the viscosity of the pure component [47].

Strong interactions result in positive values, while weak interactions between different molecules and the breaking of the self-association of the molecules lead to negative values [48,49].

**Figure 3:**
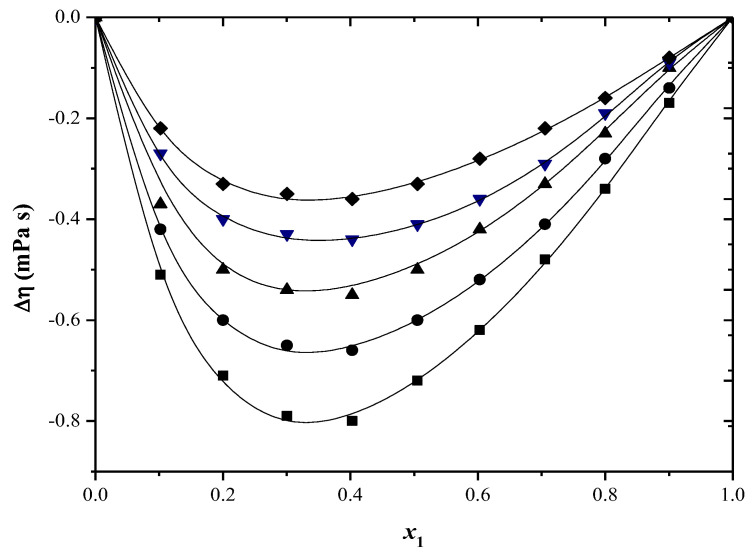
Viscosity deviation (Δ*η*) versus mole fraction for toluene (1) + 2−propanol (2) system at: ■ 293.15 K; ● 298.15 K; ▲ 303.15 K; ▼ 308.15 K; ♦ 313.15 K. The solid curve was determined with R–K equation.

**Figure 4:**
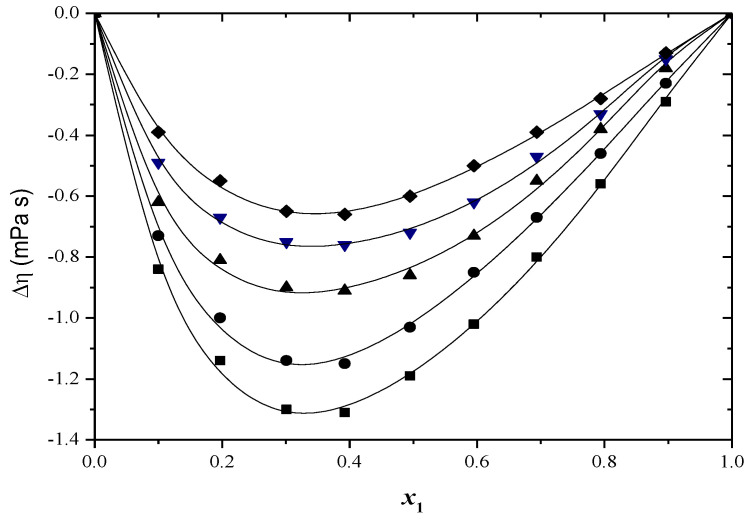
Viscosity deviation (Δ*η*) versus mole fraction for toluene (1) + 2−methyl−1−propanol (2) system at: ■ 293.15 K; ● 298.15 K; ▲ 303.15 K; ▼ 308.15 K; ♦ 313.15 K. The solid curve was determined with R–K equation.

The Δ*η* values can be seen in Table S5 and Figure 3 and Figure 4. Negative deviation values in viscosity have been observed at all the temperatures examined for both systems.

An increase in temperature leads to a decrease in viscosity deviation values. Increasing temperature reduces the self-association of alcohol and intermolecular association of the different components due to higher thermal energy. This results in decreasing negative viscosity deviation values with higher temperatures [50].

**Figure 5:**
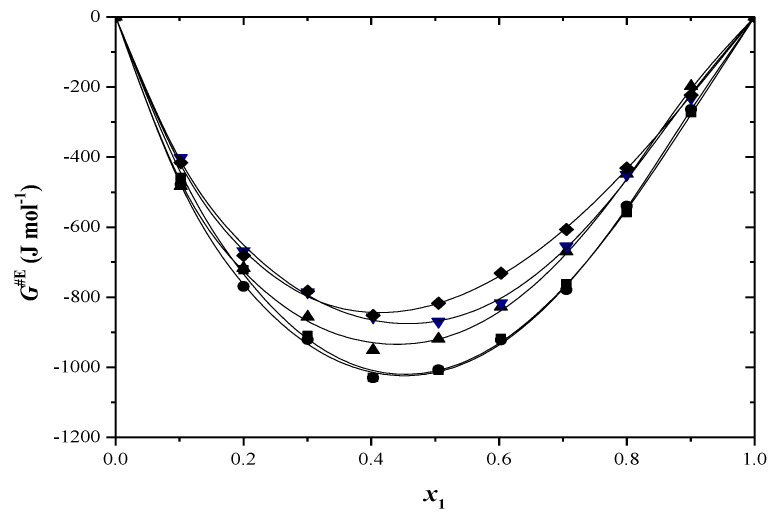
Excess Gibbs energy (Δ*G*^#E^) versus mole fraction for toluene (1) + 2−propanol (2) system at: ■ 293.15 K; ● 298.15 K; ▲ 303.15 K; ▼ 308.15 K; ♦ 313.15 K. The solid curve was determined with R–K equation.

**Figure 6:**
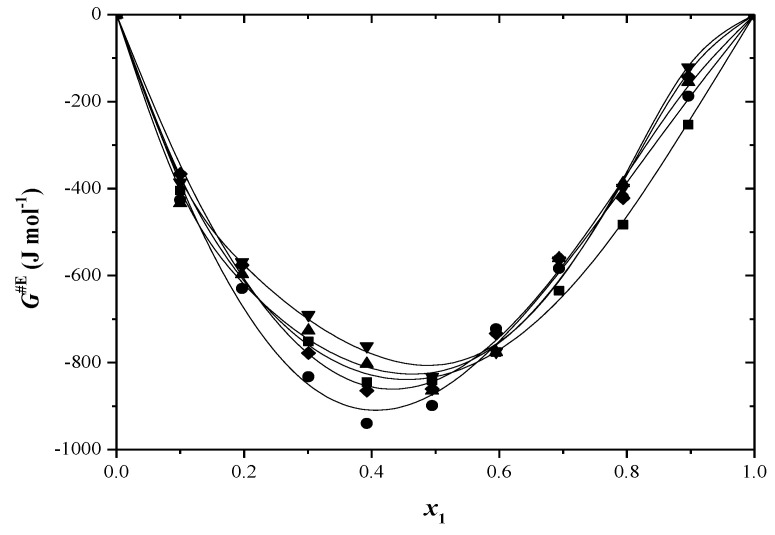
Excess Gibbs energy (Δ*G*^#E^) versus mole fraction for toluene (1) + 2−methyl−1−propanol (2) system at: ■ 293.15 K; ● 298.15 K; ▲ 303.15 K; ▼ 308.15 K; ♦ 313.15 K. The solid curve was determined with R–K equation.

The $∆G^{\neq E}$ parameter can be seen as a reliable indicator for identifying the existence of a molecular interactions [51]. For binary mixtures in which specific interactions between the molecules occur, positive values of $∆G^{\neq E}$ are obtained [52]. The experimental values of $∆G^{\neq E}$ are presented in Figure 5 and Figure 6 and Table S6.

The dispersion forces are predominant in these systems, which leads to negative values for $∆G^{\neq E}$ for all binary solutions and over the entire temperature range. The increase in temperature does not show a constant variation in these values.

The parameters and standard deviations *σ* (Equation (5)) of *V*^E^, Δ*η*, and Δ*G*^#E^ calculated by the Redlich–Kister equation were listed in Table S7.

Based on the information in Table S7, the *σ* was less than 0.025 for *V*^E^ and Δ*η* in both systems, while the maximum standard deviation for Δ*G*^#E^ was 31.2, showing that the Redlich–Kister equation was successful in representing the excess properties.

### 2.4. Apparent and Partial Molar Volumes

The apparent molar volume shows the change in volume of the solution per mole of component added when all the components are added to the solution. The partial molar volume of a liquid is the change in volume of the solution when one mole of component is added to a large excess of solution such that the composition remains unchanged. Apparent and partial molar volumes provide information about the types of interactions that occur between the components of liquid solutions [53].

The apparent molar volumes *V*_ϕ,1_ and *V*_ϕ,2_ (Equations (20) and (21)) and the partial molar volumes $\bar{V_{1}}$ and $\bar{V_{2}}$ (Equations (22) and (23)) were determined using the following formulas [54]:

(20) $$V_{\phi ,1}=\frac{x_{2}M_{2}}{x_{1}}\frac{\rho_{2}-\rho_{m}}{\rho_{2}\rho_{m}}+\frac{M_{1}}{\rho_{m}}$$

(21) $$V_{\phi ,2}=\frac{x_{1}M_{1}}{x_{2}}\frac{\rho_{1}-\rho_{m}}{\rho_{1}\rho_{m}}+\frac{M_{2}}{\rho_{m}}$$

(22) $$\bar{V_{1}}=V^{E}+V_{1}^{0}+(1-x_{1}){\left(\partial V^{E}/\partial x_{1}\right)}_{p,T}$$

(23) $$\bar{V_{2}}=V^{E}+V_{2}^{0}-x_{1}{\left(\partial V^{E}/\partial x_{1}\right)}_{p,T}$$

where $V_{1}^{0}$ and $V_{2}^{0}$ are the molar volumes of pure components. The ${\left(\partial V^{E}/\partial x_{1}\right)}_{p,T}$ value in Equations (22) and (23) was obtained by the differentiation of Equation (19) and the following equations:

(24) $$\bar{V_{1}}=V_{1}^{0}+x_{2}^{2}\sum_{k=0}^{3}a_{k}{\left(2x_{1}-1\right)}^{k}-2x_{1}x_{2}^{2}\sum_{k=1}^{3}a_{k}{\left(2x_{1}-1\right)}^{k-1}$$

(25) $$\bar{V_{2}}=V_{2}^{0}+x_{1}^{2}\sum_{k=0}^{3}a_{k}{\left(2x_{1}-1\right)}^{k}+2x_{1}^{2}x_{2}\sum_{k=1}^{3}a_{k}{\left(2x_{1}-1\right)}^{k-1}$$

The *V*_ϕ,1_ and *V*_ϕ,2_ values obtained at T = 293.15–313.15 K can be found in Tables S8 and S9. Our results indicate that as the toluene concentration rises, the *V*_ϕ,1_ values increase while the *V*_ϕ,2_ values decrease. An increase in the apparent molar volume of a solution suggests a reduction in the solute–solute interaction and an increase in the solute–solvent interaction [53]. Increasing the temperature in both systems results in an increase in the values of *V*_ϕ,1_ and *V*_ϕ,2_. The $\bar{V_{1}}$ and $\bar{V_{2}}$ values can be found in Tables S10 and S11. Increasing the toluene concentration causes the $\bar{V_{1}}$ values to decrease and the $\bar{V_{2}}$ values to rise. The positive $\bar{V}$ values suggest that the intermolecular interactions and packing effects play a key role in the binary solution. The partial molar volume values of the binary solutions were higher than the molar volumes of the pure components, which suggests that the combination of toluene and alcohol leads to an increase in the volume of the binary systems studied in this work. $\bar{V_{1}}$ and $\bar{V_{2}}$ increase as the temperature increases, probably due to the decrease in intermolecular interactions caused by the higher temperature.

### 2.5. Prigogine–Flory–Patterson (PFP) Theory

The molar excess volume experimental results were utilized to assess the validity of the PFP theories [55,56]. The PFP theory explains the thermodynamic behavior of liquid mixtures by focusing on the following three key contributions: the interactional contribution ($V_{int}^{E}$), which is linked to the interactional parameter $\chi_{12}$, the free volume contribution ($V_{FV}^{E}$), and the internal pressure contribution ($V_{p^{*}}^{E}$).

The excess molar volume given by the PFP theory is obtained through the following equation:

(26) $$\frac{V^{E}}{x_{1}V_{1}^{*}+x_{2}V_{2}^{*}}=\frac{\left({\tilde{v}}^{1/3}-1\right){\tilde{v}}^{2/3}}{\left[\left(4/3\right){\tilde{v}}^{-1/3}-1\right]}\psi_{1}\theta_{2}\frac{\chi_{12}}{p_{1}^{*}}-\frac{{\left({\tilde{v}}_{1}-{\tilde{v}}_{2}\right)}^{2}\left[\left(\frac{14}{9}\right){\tilde{v}}^{-\frac{1}{3}}-1\right]}{\left[\left(\frac{4}{3}\right){\tilde{v}}^{-\frac{1}{3}}-1\right]\tilde{v}}\psi_{1}\psi_{2}+\frac{\left({\tilde{v}}_{1}-{\tilde{v}}_{2}\right)\left(p_{1}^{*}-p_{2}^{*}\right)}{\psi_{1}p_{2}^{*}+\psi_{2}p_{1}^{*}}\psi_{1}\psi_{2}$$

Here, $\tilde{\nu }$, $p^{*}$, $\psi_{i}$, $\varphi_{i}$, and $\theta_{i}$ are the reduced volume, characteristic pressure, contact energy fraction, hard core volume fraction, and surface site fraction, which were calculated with relation to the following equations:

(27) $$\tilde{\nu }={\left(\frac{\alpha T}{3\left(1+\alpha T\right)}+1\right)}^{3}$$

(28) $$p^{*}=\frac{\alpha }{k_{T}}T{\tilde{\nu }}^{2}$$

(29) $$\psi_{1}=1-\psi_{2}=\varphi_{1}p_{1}^{*}/\left(\varphi_{1}p_{1}^{*}+\varphi_{2}p_{2}^{*}\right)$$

(30) $$\varphi_{2}=1-\varphi_{1}=x_{2}V_{2}^{*}/\left(x_{1}V_{1}^{*}+x_{2}V_{2}^{*}\right)$$

(31) $$\theta_{2}=1-\theta_{1}=\varphi_{2}V_{2}^{*1/3}/\left(\varphi_{1}V_{1}^{*1/3}+\varphi_{2}V_{2}^{*1/3}\right)$$

The various parameters in Equation (26) are calculated using Flory’s theory [57,58] and can be found in Table 5 and Table 6. By matching the experimental excess molar volume values for equimolar solutions, the interaction parameter $\chi_{12}$ was calculated to fit the theory.

Table 7 displays the values for the contribution to *V*^E^ values (interactional contribution, free volume contribution, and internal pressure effect) in equimolar mixtures. The positive values of $\chi_{12}$ are observed in the binary mixtures that were studied. An evaluation of the three contributions demonstrates that the $V_{int}^{E}$ is notable for being the most significant. The $V_{FV}^{E}$ is positive for both systems, whereas $V_{p^{*}}^{E}$ is negative for the toluene + 2-propanol system and positive for the toluene + 2-methyl-1-propanol system.

The results in Table 7 reveal that ADD (Equation (13)) values in the predictions of the excess molar volume for toluene + 2-propanol are less than 1.9% and less than 1.0% for toluene + 2-methyl-1-propanol system. These values indicate the validity of the PFP theory. Figures S7 and S8 show the comparison between the *V*^E^ values calculated from the PFP theory with the experimental values depending on the composition. As these figures show, the agreement between the *V*^E^ values calculated from PFP theory and experimental *V*^E^ data is almost good, except in the composition range *x*_1_ < 0.5 for both the systems and all temperatures.

### 2.6. FTIR Spectra

FTIR spectroscopy is used to establish the molecular structure of the substances, as well as the qualitative and quantitative analysis and study of the intermolecular interactions. An important point in the analysis of the structure of organic compounds using this instrumental method is the study of the hydrogen bonds.

**Figure 7:**
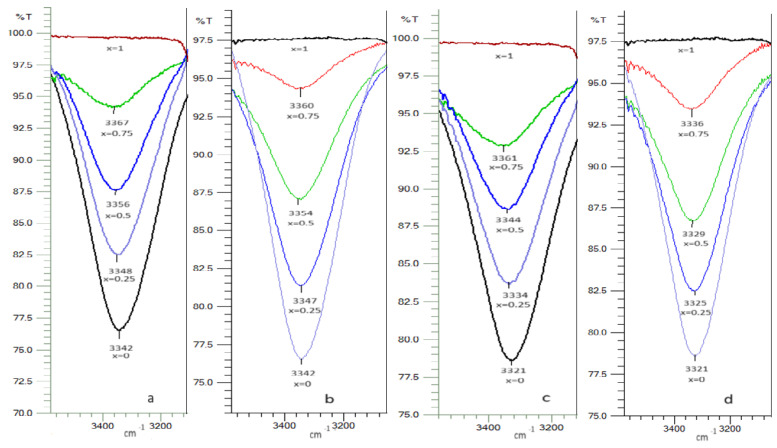
FTIR spectra of binary mixture at different mole fraction (x) and at room temperature: (**a**) toluene (*x*) − 2-propanol, (**b**) *n*-heptane (*x*) − 2-propanol, (**c**): toluene (*x*) − 2-methyl-1-propanol, (**d**): *n*-heptane (*x*) − 2−methyl-1-propanol.

As shown in Figure 7a–d, the pure toluene, pure *n*-heptane, pure 2-propanol, pure 2-methyl-1-propanol, and the binary mixtures of toluene or *n*-heptane with 2-propanol or 2-methyl-1-propanol with the different mole fractions were studied with FTIR (a: toluene-2-propanol, b: *n*-heptane-2-propanol, c: toluene -2-methyl-1-propanol, d: *n*-heptane-2-methyl-1-propanol).

Alcohols present a sharp, characteristic, and unassociated νOH band from 3600 to 3650 cm^−1^. Due to the formation of hydrogen bonds that leads to the association of alcohol molecules, a broad band appears in the IR spectrum, the associated νOH band, at 3200–3400 cm^−1^. The difference between the frequency of the unassociated νOH band and the associated νOH band is a measure of the strength of the hydrogen bonds [64].

From the plotted spectra (Figure 7a–d), it can be seen that once the alcohol (2-propanol, or 2-methyl-1-propanol) solutions are diluted with solvent (toluene or *n*-heptane), the associated νOH band moves to a higher wave number, which shows that the strength of the H bond decreases. This may be due to the alcohol dilution effect as well as the alcohol–solvent interactions. To highlight this, we used a non-polar solvent (*n*-heptane) and toluene. For the solutions, toluene + 2-propanol and *n*-heptane + 2-propanol, at identical concentrations, the displacement of the associated νOH band to a higher wave number is greater in the case of the toluene + 2-propanol solutions, which shows the existence of a weaker H bond in these solutions (Figure 7a,b). The toluene + 2-methyl-1-propanol and *n*-heptane + 2-methyl-1-propanol solutions show the same behavior, but the effect is more important (Figure 7c,d).

The existence of a weaker H bond in the alcohol solutions with toluene compared to the alcohol solutions with *n*-heptane is caused by the interaction of the π electrons of the aromatic nucleus of toluene with the proton of the OH bond in alcohol. This effect is greater in solutions with 2-methyl-1-propanol than in solutions with 2-propanol in accordance with the greater strength of this alcohol. This type of intermolecular interaction was observed by other authors in similar systems [13,65,66].

IR studies show that as the amount of toluene in alcohol solutions increases, the strength of the hydrogen bond between the alcohol molecules decreases, as well as the existence of weak toluene–alcohol intermolecular interactions, slightly stronger in the toluene–2-methyl-1-propanol system. The presence of weak interactions between the toluene and alcohol molecules is in agreement with the positive values of the excess molar volumes and the negative values of the viscosity deviation for all the systems studied in this paper.

## 3. Materials and Methods

### 3.1. Materials

Table 8 includes all details of the chemical samples. All determinations were taken at a pressure of 100 kPa, which was determined in our laboratory with an accuracy of ±2 kPa.

### 3.2. Experimental Analysis

Analytical balance Adventurer AX 224M with a precision ±10^−4^ g was used for weighing the samples.

Densities were measured with a digital densimeter (model DA 650 KEM—Kyoto Electronics manufacturing, Tokyo, Japan) at P = 100 kPa. The procedures for measuring the density and viscosity were explained earlier [67]. The uncertainty for the mole fraction of the mixtures was below 3 × 10^−4^. The combined expanded uncertainty of the densities was calculated to be 0.0006 g·cm^−3^, and the expanded uncertainties in the *V*^E^ were estimated to be 0.08 cm^3^·mol^−1^ (0.95 of confidence).

Viscosities of the pure compounds and of the binary solutions were determined with an Ubbelohde kinematic, viscosity measuring unit ViscoClock (Schott-Gerate GmbH, Mainz, Germany) that was kept in a vertical position in a thermostatic bath (model TV 2000 Tamson, Bleiswijk, The Netherlands). The temperature was controlled with a precision of ±0.05 K.

The kinematic viscosity was calculated using the following equation:

(32) $$\nu =At-\left(B/t\right)$$

where *ν* is the kinematic viscosity, *t* is the flow time, and *A* and *B* are characteristic constants of the viscometer. The constants *A* and *B* were determined by taking doubly distilled water and benzene (Merck, Boston, MA, USA, mole fraction purity ≥ 0.995) as the calibrating liquids. The accuracy of time measurement is ±0.01 s.

Equation (33) was used to calculate the dynamic viscosity:

(33) η=νρ

where *ρ* is the density.

The overall expanded uncertainty of dynamic viscosity was calculated as 0.02 mPa·s. The expanded uncertainties in the ∆η were calculated to be 0.04 mPa·s with a confidence level of 0.95.

### 3.3. Spectral Analyses

FTIR spectra were carried out at P = 100 kPa and room temperature. FTIR spectra of binary solutions at mole fractions *x*_1_ = 0.0, 0.25, 0.50, 0.75, and 1.0 was recorded using the IRAFINITY spectrometer (Schimatzu, Columbia, MD, USA), with a wavelength range of 4000–500 cm^−1^.

## 4. Conclusions

Measurements of density and viscosity for the toluene + 2-propanol and toluene + 2-methyl-1-propanol systems were determined at T = 293.15–313.15 K and P = 100 kPa. The density of binary solutions was correlated using the Emmerling et al. and the Gonzalez-Olmos–Iglesias equations. The results indicate that the equation by Emmerling et al. is the best to correlate the density for toluene + 2-methyl-1-propanol system, while for toluene + 2-propanol, both proposed equations are proper to correlate the density with composition and temperature. Both the systems had positive *V*^E^ values and negative Δ*η* values in their calculations. All binary solutions showed negative values for the excess Gibbs activation energy. Models from Wilson, Noda–Ishida, Eyring–NRTL, Eyring–Van Laar, and Eyring–Margules were used to correlate the viscosity. The results indicate that the Eyring–Margules model is the best for describing the viscosities of the binary mixtures. The activation energies of the viscous flow were determined. The values of $∆G^{\neq }$ and $∆H^{\neq }$ are positive for both binary systems. The value of $∆S^{\neq }$ is positive for the alcohols and alcohol-rich solutions but negative for the toluene and toluene-rich solutions. The experimental results of *V*^E^ have been used to test the applicability of the Prigogine–Flory–Patterson theory. The values of the percentage of absolute average deviation obtained suggest the validity of this theory. The FTIR spectral analysis was utilized to examine and comprehend the intermolecular interactions in the binary systems of toluene with 2-propanol and 2-methyl-1-propanol.

## Figures and Tables

**Table 4:** Values of $∆G^{\neq }$, $∆H^{\neq }$, $∆S^{\neq }$ for the binary mixtures.

*x*_1_	∆H^≠^ (kJ/mol)	∆S^≠^ (J/mol·K)	∆G^≠^ (kJ/mol) T/(K)				
			293.15	298.15	303.15	308.15	313.15
toluene (1) + 2-propanol (2)							
0.0000	21.66	22.88	14.95	14.84	14.72	14.61	14.49
0.1020	18.83	15.77	14.21	14.13	14.05	13.97	13.89
0.2001	17.03	11.42	13.68	13.62	13.57	13.51	13.45
0.3004	14.22	3.35	13.24	13.22	13.20	13.19	13.17
0.4030	11.85	−3.40	12.85	12.87	12.88	12.90	12.92
0.5051	10.53	−7.08	12.61	12.64	12.68	12.71	12.75
0.6029	9.51	−9.99	12.44	12.49	12.54	12.59	12.64
0.7052	8.47	−13.17	12.33	12.40	12.46	12.53	12.59
0.7998	7.87	−15.13	12.30	12.38	12.46	12.53	12.61
0.9005	7.97	−14.90	12.34	12.41	12.49	12.56	12.64
1.0000	7.69	−15.87	12.35	12.43	12.50	12.58	12.66
toluene (1) + 2-methyl-1-propanol (2)							
0.0000	21.41	16.33	16.62	16.54	16.46	16.37	16.29
0.1000	18.95	10.87	15.77	15.71	15.66	15.60	15.55
0.1969	17.76	8.81	15.18	15.13	15.09	15.05	15.00
0.3008	15.93	4.67	14.56	14.54	14.51	14.49	14.47
0.3922	14.33	0.86	14.07	14.07	14.06	14.06	14.06
0.4947	13.58	−0.20	13.64	13.64	13.64	13.64	13.64
0.5952	12.30	−3.47	13.31	13.33	13.35	13.37	13.38
0.6936	10.22	−9.61	13.04	13.09	13.13	13.18	13.23
0.7941	9.18	−12.26	12.77	12.83	12.90	12.96	13.02
0.8965	7.09	−18.63	12.55	12.65	12.74	12.83	12.93
1.0000	7.69	−15.87	12.35	12.43	12.50	12.58	12.66

**Table 5:** Characteristic parameters of the Flory theory for pure compounds.

Component	10^4^·*α*/K^−1^	*k*_T_/10^4^·MPa^−1^	$\tilde{v}$	V*/cm^3^ mol^−1^	10^6^·p*/Jcm^−3^	T*/K	$\tilde{T}$
293.15 K							
toluene	9.84	8.715 [59]	1.24	85.68	509.76	5238.74	0.056
2-propanol	10.75	10.81 [60]	1.26	60.72	462.29	4990.87	0.059
2-methyl- 1-propanol	11.38	9.828 [11]	1.27	72.74	548.86	4843.24	0.060
298.15 K							
toluene	9.92	9.023 [59]	1.25	85.76	508.95	5254.95	0.057
2-propanol	10.99	11.3 [61]	1.27	60.59	465.97	4972.21	0.060
2-methyl- 1-propanol	11.48	10.26 [62]	1.28	72.77	544.26	4861.14	0.061
303.15 K							
toluene	10.00	9.349 [59]	1.25	85.84	507.57	5270.52	0.057
2-propanol	11.24	11.8 [63]	1.28	60.57	470.36	4954.06	0.061
2-methyl- 1-propanol	11.59	10.649 [11]	1.28	72.93	543.26	4877.43	0.062
308.15 K							
toluene	10.08	9.694 [59]	1.26	85.92	505.65	5287.15	0.058
2-propanol	11.49	12.32 [51]	1.29	60.51	474.50	4938.74	0.062
2-methyl- 1-propanol	11.69	11.086 [62]	1.29	72.99	539.80	4896.38	0.063
313.15 K							
toluene	10.17	10.06 [59]	1.26	86.00	503.80	5301.47	0.059
2-propanol	11.74	15.117 [11]	1.29	60.42	406.97	4926.06	0.064
2-methyl-1-propanol	11.79	11.541 [11]	1.29	73.10	536.17	4915.81	0.064

**Table 6:** Parameters of the equimolar solutions of the PFP theory.

*T*/K	*φ*_2_	*θ*_2_	$\tilde{v}$	$\tilde{T}$	*χ*_12_·10^6^/Jcm^−3^	*ψ*_1_
toluene (1) + 2-propanol (2)						
293.15	0.415	0.334	1.26	0.059	69.82	0.6088
298.15	0.414	0.333	1.26	0.060	67.24	0.6072
303.15	0.414	0.332	1.27	0.060	62.70	0.6046
308.15	0.413	0.331	1.28	0.061	60.44	0.6021
313.15	0.413	0.330	1.28	0.062	59.02	0.6379
toluene (1) + 2-methyl-1-propanol (2)						
293.15	0.459	0.419	1.26	0.059	39.29	0.5225
298.15	0.459	0.419	1.27	0.060	37.25	0.5243
303.15	0.459	0.419	1.27	0.061	35.16	0.5237
308.15	0.459	0.419	1.28	0.061	32.97	0.5244
313.15	0.459	0.419	1.28	0.062	31.18	0.5250

**Table 7:** Experimental and calculated excess volumes and values of the contributions to *V*^E^ at *x* = 0.5.

*T*/K	*V*^E^_exp._/ cm^3^ mol^−1^	*V*^E^_PFP_/ cm^3^ mol^−1^	*V*^E^_int._/ cm^3^ mol^−1^	*V*^E^_FV_/ cm^3^ mol^−1^	*V*^E^_p*_/ cm^3^ mol^−1^	ADD%
toluene (1) + 2-propanol (2)						
293.15	0.755	0.768	0.808	0.009	−0.031	1.7
298.15	0.742	0.755	0.801	0.012	−0.034	1.7
303.15	0.707	0.719	0.769	0.016	−0.034	1.7
308.15	0.700	0.712	0.765	0.021	−0.032	1.7
313.15	0.660	0.673	0.818	0.026	−0.119	1.9
toluene (1) + 2-methyl-1-propanol (2)						
293.15	0.550	0.555	0.538	0.028	0.045	0.9
298.15	0.535	0.540	0.528	0.029	0.041	0.9
303.15	0.522	0.527	0.514	0.030	0.043	0.9
308.15	0.505	0.509	0.499	0.031	0.042	0.8
313.15	0.492	0.497	0.488	0.032	0.041	1.0

**Table 8:** Specification of chemical compounds.

Chemical Name	Molecular Formula	Source	CAS	Mass Fraction Purity	Water Content	Purification
Toluene	C7H8	Lach:ner	108-88-3	≥99.92%	≤0.08%	None
2-propanol	C3H8O	Merck	67-63-0	≥99.7%	≤0.3%	None
2-methyl-1-propanol	C4H10O	Chemical Company	78-83-1	≥99.7%	≤0.3%	None

## Data Availability

Data are contained within the article and Supplementary Materials.

## Supplementary Materials

The following supporting information can be downloaded at: https://www.mdpi.com/article/10.3390/molecules29194706/s1, Figure S1: Density (*ρ*) versus mole fraction for toluene (1) + 2-propanol (2) system at: ■ 293.15 K; ● 298.15 K; ▲ 303.15 K; ▼ 308.15 K; ♦ 313.15 K; Figure S2: Density (*ρ*) versus mole fraction for toluene (1) + 2-methyl-1-propanol (2) system at: ■ 293.15 K; ● 298.15 K; ▲ 303.15 K; ▼ 308.15 K; ♦ 313.15 K; Figure S3: Viscosity (*η*) versus mole fraction for toluene (1) + 2-propanol (2) system at: ■ 293.15 K; ● 298.15 K; ▲ 303.15 K; ▼ 308.15 K; ♦ 313.15 K; Figure S4: Viscosity (*η*) versus mole fraction for toluene (1) + 2-methyl-1-propanol (2) system at: ■ 293.15 K; ● 298.15 K; ▲ 303.15 K; ▼ 308.15 K; ♦ 313.15 K; Figure S5: Gibbs activation energy ($∆G^{\neq }$) versus mole fraction for toluene (1) + 2-propanol (2) system at: ■ 293.15 K; ● 298.15 K; ▲ 303.15 K; ▼ 308.15 K; ♦ 313.15 K; Figure S6: Gibbs activation energy ($∆G^{\neq }$) versus mole fraction for toluene (1) + 2-methyl-1-propanol (2) system at: ■ 293.15 K; ● 298.15 K; ▲ 303.15 K; ▼ 308.15 K; ♦ 313.15 K; Figure S7: Excess molar volumes (*V*^E^) versus mole fraction for toluene (1) + 2-propanol (2) system at: ■ 293.15 K; ● 298.15 K; ▲ 303.15 K; ▼ 308.15 K; ♦ 313.15 K. The solid curve was calculated from PFP theory; Figure S8: Excess molar volumes (*V*^E^) versus mole fraction for toluene (1) + 2-propanol (2) system at: ■ 293.15 K; ● 298.15 K; ▲ 303.15 K; ▼ 308.15 K; ♦ 313.15 K. The solid curve was calculated from PFP theory; Table S1: Values of parameters at T = 293.15–313.15 K for the Emmerling et al. and Gonzales-Olmos–Iglesias models and standard deviations^1^; Table S2: Isobaric thermal expansion coefficient values ($10^{4}\alpha_{p}$/K^−1^) as a functions of mole fraction at T = 293.15–313.15 K and P = 100 kPa; Table S3: Values of parameters for the relations of Wilson, Noda and Ishida and Eyring-NRTL and average absolute deviation at T = 293.15–313.15 K; Table S4: Excess molar volume (*V*^E^/cm^3^mol^−1^) of toluene (1) + 2-propanol (2) and toluene (1) + 2-methyl-1-propanol (2) systems at (293.15–318.15) K; Table S5: Viscosity deviation (Δ*η*/mPa s) of toluene (1) + 2-propanol (2) and toluene (1) + 2-methyl-1-propanol (2) systems at (293.15–318.15) K; Table S6: Gibbs activation energy (Δ*G*^#E^/J mol^−1^) of toluene (1) + 2-propanol (2) and toluene (1) + 2-methyl-1-propanol (2) systems at (293.15–318.15) K; Table S7: Polynomial coefficients and standard deviations (*σ*) for the binary systems at T = 293.15–313.15 K; Table S8: Apparent molar volumes *V*_ϕ,1_(cm^3^ mol^−1^) for binary systems at T = 293.15–313.15 K; Table S9: Apparent molar volumes *V*_ϕ,2_ (cm^3^ mol^−1^) for binary systems at T = 293.15–313.15 K; Table S10: Partial molar volumes $\bar{V_{1}}$(cm^3^ mol^−1^) for binary systems at T = 293.15–313.15 K; Table S11: Partial molar volumes $\bar{V_{2}}$(cm^3^ mol^−1^) for binary systems at T = 293.15–313.15 K.
