# Supplementary material for: Excess Thermodynamic Properties and FTIR Studies of Binary Mixtures of Toluene with 2-Propanol or 2-Methyl-1-Propanol
Source: Molecules. 2024 Oct 4;29(19):4706. doi: 10.3390/molecules29194706 (PMC11477799; doi:10.3390/molecules29194706)
Supplement: Supplementary file 1 [file molecules-29-04706-s001.zip › molecules-3219016-supplementary.pdf]

Excess Thermodynamic Properties and FTIR Studies of Binary Mixtures of Toluene with 2-Propanol or 2-Methyl-1-propanol

Maria Magdalena Naum * and Vasile Dumitrescu

Chemistry Department, Petroleum and Gas University of Ploiesti, 100680 Ploiesti, Romania
* Correspondence: maria.budeanu@upg-ploiesti.ro

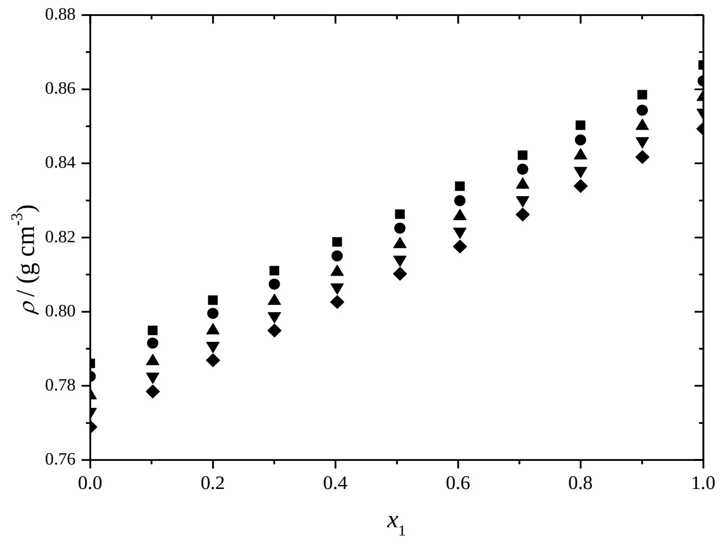

Figure S1. Density (ρ) versus mole fraction for toluene (1) + 2-propanol (2) system at: ■ 293.15 K; ● 298.15 K; ▲ 303.15 K; ▼ 308.15 K; ♦ 313.15K.

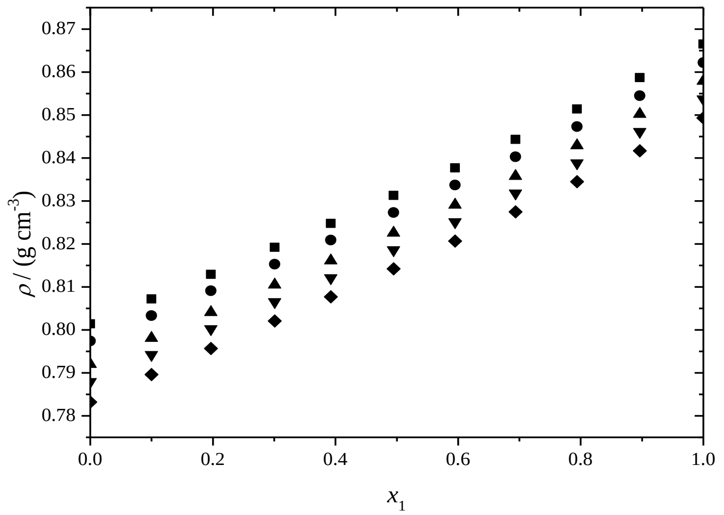

Figure S2. Density (ρ) versus mole fraction for toluene (1) + 2-methyl-1-propanol (2) system at: ■ 293.15 K; ● 298.15 K; ▲ 303.15 K; ▼ 308.15 K; ♦ 313.15K.

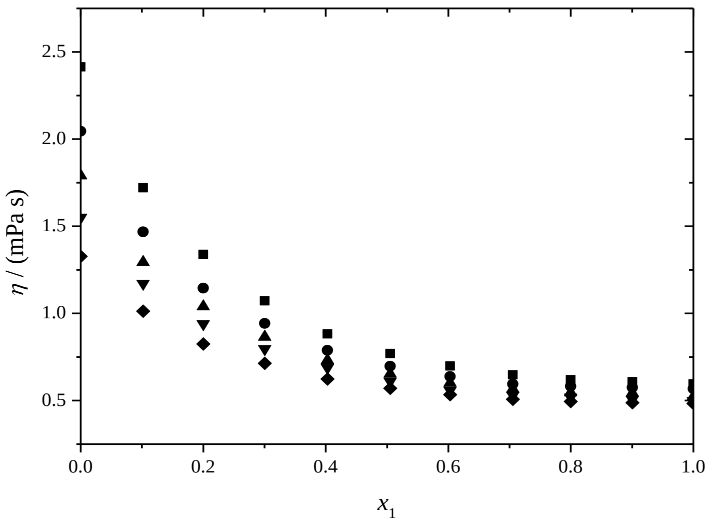

Figure S3. Viscosity (η) versus mole fraction for toluene (1) + 2-propanol (2) system at: ■ 293.15 K; ● 298.15 K; ▲ 303.15 K; ▼ 308.15 K; ♦ 313.15K.

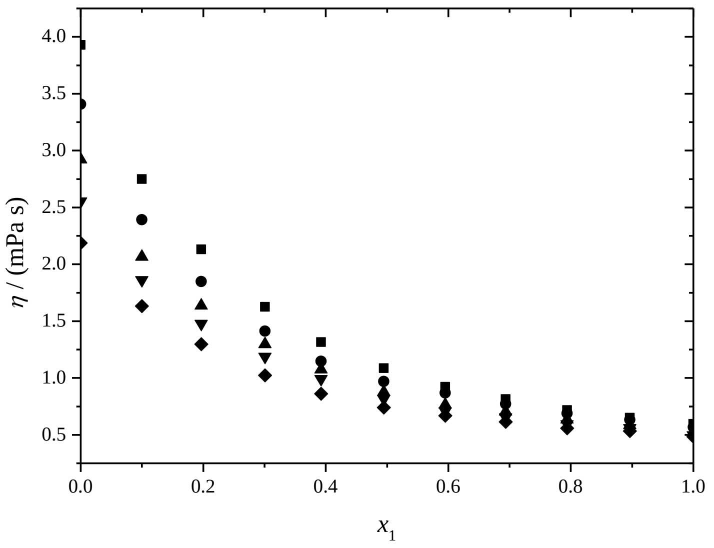

Figure S4. Viscosity (η) versus mole fraction for toluene (1) + 2-methyl-1-propanol (2) system at: ■ 293.15 K; ● 298.15 K; ▲ 303.15 K; ▼ 308.15 K; ♦ 313.15K.

Table S1. Values of parameters at T = 293.15-313.15 K for the Emmerling *et al.* and Gonzales-Olmos-Iglesias models and standard deviations[1]

toluene (1) + 2-propanol (2)		
Emmerling *et al.*		
$A_1 = 1.0919$	$B_1 = -6.8334 \cdot 10^{-4}$	$C_1 = -2.9209 \cdot 10^{-7}$
$A_2 = 0.9078$	$B_2 = 1.2941 \cdot 10^{-5}$	$C_2 = -1.4603 \cdot 10^{-6}$
$P_1 = 0.1582$	$P_2 = -0.0013$	$P_3 = 2.4278 \cdot 10^{-6}$
$P_4 = -0.1637$	$P_5 = 0.0010$	$P_6 = -1.7754 \cdot 10^{-6}$
$P_7 = 0.0108$	$P_8 = -1.8072 \cdot 10^{-4}$	$P_9 = 6.0428 \cdot 10^{-7}$
	$10^4 \cdot \sigma = 2.43$	
Gonzalez-Olmos-Iglesias		
$A_{00} = 0.9200$	$A_{01} = -6.8766 \cdot 10^{-5}$	$A_{02} = -1.3141 \cdot 10^{-6}$
$A_{10} = 0.3182$	$A_{11} = -0.0018$ A_{21}	$A_{12} = 3.3342 \cdot 10^{-6}$
$A_{20} = -0.1577$	$= 0.0012$	$A_{22} = -2.4228 \cdot 10^{-6}$
	$10^4 \cdot \sigma = 2.44$	
toluene (1) + 2-methyl-1-propanol (2)		
Emmerling *et al.*		
$A_1 = 1.0865$	$B_1 = -6.4870 \cdot 10^{-4}$	$C_1 = -3.4697 \cdot 10^{-7}$
$A_2 = 1.0517$	$B_2 = -7.9262 \cdot 10^{-4}$	$C_2 = -2.0724 \cdot 10^{-7}$
$P_1 = -0.1120$	$P_2 = 5.3926 \cdot 10^{-4}$	$P_3 = -6.7251 \cdot 10^{-7}$
$P_4 = 0.1680$	$P_5 = -0.0011$	$P_6 = 1.6898 \cdot 10^{-6}$
$P_7 = -0.2611$	$P_8 = 0.0015$	$P_9 = -2.2181 \cdot 10^{-6}$
	$10^4 \cdot \sigma = 2.63$	
Gonzalez-Olmos-Iglesias		
$A_{00} = 1.0319$	$A_{01} = -6.6954 \cdot 10^{-4}$	$A_{02} = -3.9368 \cdot 10^{-7}$
$A_{10} = -0.0526$	$A_{11} = 5.2462 \cdot 10^{-4}$	$A_{12} = -5.6478 \cdot 10^{-7}$
$A_{20} = 0.1118$	$A_{21} = -5.3740 \cdot 10^{-4}$	$A_{22} = 6.6942 \cdot 10^{-7}$
	$10^4 \cdot \sigma = 3.75$	

[1]Units: A_i, P_1, P_4, P_7, A_{00}, A_{10}, A_{20}, σ: $g \cdot cm^{-3}$; B_i, P_2, P_5, P_8, A_{01}, A_{11}, A_{21}: $g \cdot cm^{-3} K^{-1}$;
C_i, P_3, P_6, P_9, A_{02}, A_{12}, A_{22}: $g \cdot cm^{-3} K^{-2}$

Table S2. Isobaric thermal expansion coefficient values ($10^4\alpha_p$ / K^{-1}) as a functions of mole fraction at T = 293.15-313.15 K and P = 100 kPa

x_1	$10^4\alpha_p$ / K^{-1}				
	**293.15**	**298.15**	**303.15**	**308.15**	**313.15**
toluene (1) + 2-propanol (2)					
0.0000	10.75	10.99	11.24	11.49	11.74
0.1020	10.23	10.42	10.63	10.83	11.03
0.2001	10.16	10.24	10.34	10.43	10.52
0.3004	9.92	10.03	10.16	10.29	10.41
0.4030	9.83	9.94	10.06	10.19	10.31
0.5051	9.83	9.87	9.92	9.97	10.02
0.6029	9.69	9.77	9.86	9.94	10.01
0.7052	9.50	9.58	9.66	9.74	9.82
0.7998	9.60	9.68	9.76	9.84	9.93
0.9005	9.69	9.77	9.86	9.94	10.02
1.0000	9.84	9.92	10.00	10.08	10.17
toluene (1) + 2-methyl-1-propanol (2)					
0.0000	11.39	11.48	11.59	11.69	11.79
0.1000	10.90	10.99	11.1	11.19	11.29
0.1969	10.44	10.60	10.77	10.93	11.10
0.3008	10.36	10.48	10.61	10.73	10.86
0.3922	10.29	10.41	10.54	10.66	10.79
0.4947	10.25	10.34	10.43	10.52	10.61
0.5952	10.03	10.15	10.28	10.39	10.52
0.6936	9.90	9.99	10.07	10.16	10.24
0.7941	9.87	9.95	10.04	10.12	10.21
0.8965	9.74	9.86	9.97	10.09	10.21
1.0000	9.84	9.92	10.00	10.08	10.17

Table S3. Values of parameters for the relations of Wilson, Noda and Ishida and Eyring-NRTL and average absolute deviation at T = 293.15-313.15 K

Model	Parameters and ADD%	T/ (K)				
		**293.15**	**298.15**	**303.15**	**308.15**	**313.15**
toluene (1) + 2-propanol (2)						
Wilson	λ_{12}	4005.951	4566.794	5133.455	3611.582	4525.740
	λ_{21}	2211.254	2047.354	1421.499	1593.458	1134.869
	ADD%	0.68	0.66	0.70	0.60	0.32
Noda and Ishida	w_{12}	-155.841	-167.385	-182.909	-160.629	-178.729
	w_{21}	144.904	155.238	169.134	149.858	166.025
	ADD%	0.38	0.46	0.59	0.57	0.43
Eyring-NRTL[a]	τ_{12}	-1.451	-1.576	-1.908	-1.469	-1.896
	τ_{21}	0.123	0.332	1.216	0.475	1.476
	ADD%	0.43	0.47	0.64	0.57	0.30
Eyring-Van Laar	A	-1.338	-1.284	-1.072	-1.065	-0.933
	B	0.630	0.581	0.487	0.584	0.481
	ADD%	0.43	0.47	0.64	0.57	0.22
Eyring-Margules	A_{21}	-1.265	-1.194	-0.936	-0.991	-0.824
	A_{12}	-2.042	-2.089	-1.997	-1.725	-1.743
	ADD%	0.37	0.46	0.57	0.56	0.44
toluene (1) + 2-methyl-1-propanol (2)						
Wilson	λ_{12}	3553.089	5675.233	4852.346	4024.604	4498.997
	λ_{21}	1231.913	649.476	590.985	714.192	673.360
	ADD%	0.61	1.55	1.48	1.44	1.41
Noda and Ishida	w_{12}	-145.198	-191.803	-178.715	-167.098	-180.474
	w_{21}	135.727	176.718	165.670	155.710	167.590
	ADD%	0.51	0.89	1.37	1.35	0.97
Eyring-NRTL[a]	τ_{12}	-1.333	-1.938	-1.839	-1.576	-1.735
	τ_{21}	0.243	1.421	1.343	0.848	1.140
	ADD%	0.55	1.36	1.48	1.43	1.18
Eyring-Van Laar	A	-1.112	-0.972	-0.928	-0.926	-0.923
	B	0.634	0.441	0.483	0.544	0.506
	ADD%	0.55	1.24	1.48	1.43	1.21
Eyring-Margules	A_{21}	-1.060	-0.763	-0.782	-0.812	-0.766
	A_{12}	-1.683	-1.991	-1.765	-1.615	-1.721
	ADD%	0.50	0.87	1.34	1.32	0.93

[a]Eyring-NRTL as two-parameter model (α = 0.30)

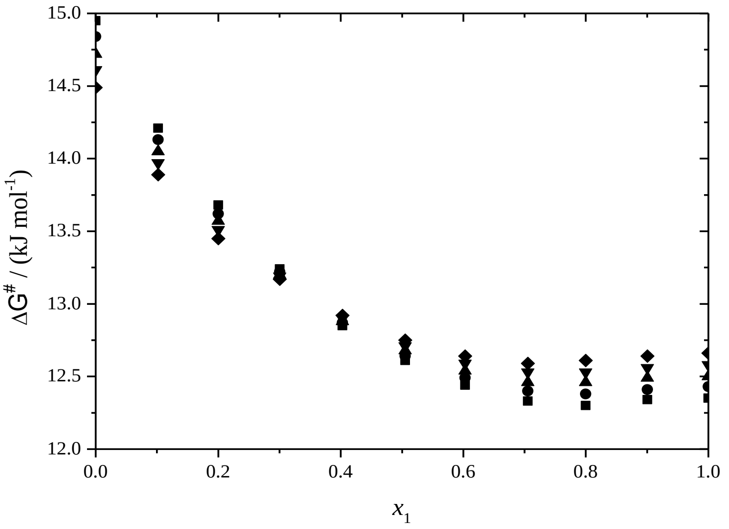

Figure S5. Gibbs activation energy (ΔG≠) versus mole fraction for toluene (1) + 2-propanol (2) system at: ■ 293.15 K; ● 298.15 K; ▲ 303.15 K; ▼ 308.15 K; ♦ 313.15K.

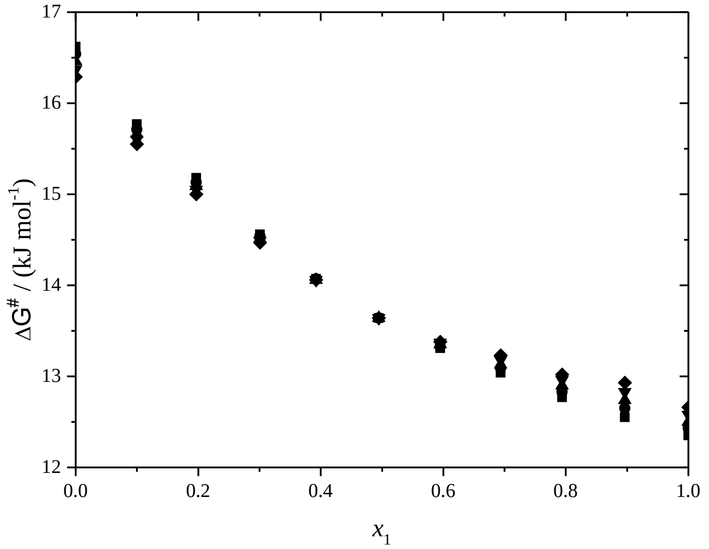

Figure S6. Gibbs activation energy (ΔG≠) versus mole fraction for toluene (1) + 2-methyl-1-propanol (2) system at: ■ 293.15 K; ● 298.15 K; ▲ 303.15 K; ▼ 308.15 K; ♦ 313.15K.

Table S4. Excess molar volume (V^E / cm^3mol^{-1}) of toluene (1) + 2-propanol (2) and toluene (1) + 2-methyl-1-propanol (2) systems at (293.15 – 318.15) K

x_1	***T* / (K)**				
	**293.15**	**298.15**	**303.15**	**308.15**	**313.15**
toluene (1) + 2-propanol (2)					
0.0000	0	0	0	0	0
0.1020	0.21	0.19	0.18	0.15	0.14
0.2001	0.38	0.37	0.34	0.31	0.29
0.3004	0.54	0.52	0.49	0.47	0.44
0.4030	0.67	0.66	0.62	0.61	0.58
0.5051	0.76	0.75	0.72	0.71	0.67
0.6029	0.77	0.76	0.73	0.72	0.68
0.7052	0.66	0.63	0.60	0.58	0.54
0.7998	0.46	0.44	0.42	0. 41	0.38
0.9005	0.25	0.24	0.23	0.22	0.21
1.0000	0	0	0	0	0
toluene (1) + 2-methyl-1-propanol (2)					
0.0000	0	0	0	0	0
0.1000	0.18	0.17	0.16	0.14	0.13
0.1969	0.33	0.30	0.28	0.26	0.24
0.3008	0.44	0.42	0.39	0.38	0.36
0.3922	0.51	0.49	0.48	0.46	0.45
0.4947	0.54	0.53	0.52	0.50	0.49
0.5952	0.55	0.54	0.53	0.51	0.50
0.6936	0.50	0.48	0.47	0.45	0.43
0.7941	0.38	0.36	0.35	0.34	0.33
0.8965	0.23	0.22	0.21	0.20	0.19
1.0000	0	0	0	0	0

Standard uncertainties: $u(p)$ = 2 kPa, $u(T)$ = 0.05 K; Expanded uncertainties: $U(\rho)$ = 0.0006 $g \cdot cm^{-3}$, $U(V^E)$ = 0.08 $cm^3 \cdot mol^{-1}$ (0.95 of confidence).

Table S5. Viscosity deviation (Δη / mPa s) of toluene (1) + 2-propanol (2) and toluene (1) + 2-methyl-1-propanol (2) systems at (293.15 – 318.15) K

	T / (K)				
x_1	**293.15**	**298.15**	**303.15**	**308.15**	**313.15**
toluene (1) + 2-propanol (2)					
0.0000	0	0	0	0	0
0.1020	-0.51	-0.42	-0.37	-0.27	-0.22
0.2001	-0.71	-0.60	-0.50	-0.40	-0.33
0.3004	-0.79	-0.65	-0.54	-0.43	-0.35
0.4030	-0.80	-0.66	-0.55	-0.44	-0.36
0.5051	-0.72	-0.60	-0.50	-0.41	-0.33
0.6029	-0.62	-0.52	-0.42	-0.36	-0.28
0.7052	-0.48	-0.41	-0.33	-0.29	-0.22
0.7998	-0.34	-0.28	-0.23	-0.19	-0.16
0.9005	-0.17	-0.14	-0.10	-0.09	-0.08
1.0000	0	0	0	0	0
toluene (1) + 2-methyl-1-propanol (2)					
0.0000	0	0	0	0	0
0.1000	-0.84	-0.73	-0.62	-0.49	-0.39
0.1969	-1.14	-1.00	-0.81	-0.67	-0.55
0.3008	-1.30	-1.14	-0.90	-0.75	-0.65
0.3922	-1.31	-1.15	-0.91	-0.76	-0.66
0.4947	-1.19	-1.03	-0.86	-0.72	-0.60
0.5952	-1.02	-0.85	-0.73	-0.62	-0.50
0.6936	-0.80	-0.67	-0.55	-0.47	-0.39
0.7941	-0.56	-0.46	-0.38	-0.33	-0.28
0.8965	-0.29	-0.23	-0.18	-0.15	-0.13
1.0000	0	0	0	0	0

Standard uncertainties: $u(p)$ = 2 kPa, $u(T)$ = 0.05 K; Expanded uncertainties: $U(\eta)$ = 0.02 mPa·s, $U(\Delta\eta)$ = 0.04 mPa·s (0.95 of confidence).

Table S6. Excess Gibbs activation energy ($\Delta G^{\#E}$ /J mol^{-1}) of toluene (1) + 2-propanol (2) and toluene (1) + 2-methyl-1-propanol (2) systems at (293.15 – 318.15) K

x_1	***T* / (K)**				
	**293.15**	**298.15**	**303.15**	**308.15**	**313.15**
toluene (1) + 2-propanol (2)					
0.0000	0	0	0	0	0
0.1020	-458.45	-477.66	-482.66	-402.67	-415.74
0.2001	-721.78	-768.90	-716.52	-668.21	-680.66
0.3004	-909.39	-920.95	-856.09	-785.55	-782.40
0.4030	-1028.54	-1030.15	-951.26	-856.90	-852.22
0.5051	-1008.91	-1007.34	-919.19	-869.61	-816.80
0.6029	-917.33	-922.03	-827.08	-816.38	-731.72
0.7052	-761.69	-778.87	-669.20	-654.94	-607.01
0.7998	-558.78	-540.26	-447.48	-450.12	-431.79
0.9005	-273.65	-264.43	-197.46	-231.76	-223.45
1.0000	0	0	0	0	0
toluene (1) + 2-methyl-1-propanol (2)					
0.0000	0	0	0	0	0
0.1000	-404.41	-426.22	-433.88	-386.22	-366.32
0.1969	-573.13	-629.99	-596.51	-569.31	-575.58
0.3008	-751.94	-832.67	-726.63	-690.22	-778.14
0.3922	-845.04	-939.74	-802.71	-762.42	-864.39
0.4947	-841.19	-898.84	-864.39	-832.36	-860.94
0.5952	-775.84	-722.41	-775.60	-775.52	-732.92
0.6936	-634.95	-583.84	-560.11	-565.11	-559.96
0.7941	-483.35	-419.79	-387.78	-398.46	-421.82
0.8965	-253.27	-187.71	-155.10	-121.35	-143.53
1.0000	0	0	0	0	0

Table S7. Polynomial coefficients and standard deviations (σ) for the binary systems at T = 293.15-313.15 K

	Parameters and σ				
	a_0	a_1	a_2	a_3	σ
toluene (1) + 2-propanol (2)					
293.15 K					
V^E / (cm^3 · mol^{-1})	3.02	0.94	-0.92	-1.14	0.012
$\Delta\eta$ / (mPa·s)	-2.89	1.67	-1.17	0.90	0.010
$\Delta G^{\neq E}$ (J · mol^{-1})	-4043.72	774.88	91.88	519.01	11.93
298.15 K					
V^E / (cm^3 · mol^{-1})	2.97	0.86	-1.12	-0.97	0.015
$\Delta\eta$ / (mPa·s)	-2.41	1.30	-0.97	0.95	0.006
$\Delta G^{\neq E}$ (J · mol^{-1})	-4058.32	767.14	-72.16	1032.12	10.39
303.15 K					
V^E / (cm^3 · mol^{-1})	2.83	0.91	-1.13	-1.03	0.016
$\Delta\eta$ / (mPa·s)	-1.98	1.08	-0.84	1.07	0.009
$\Delta G^{\neq E}$ (J · mol^{-1})	-3686.21	851.83	-68.34	1465.14	13.60
308.15 K					
V^E / (cm^3 · mol^{-1})	2.80	0.89	-1.40	-0.76	0.016
$\Delta\eta$ / (mPa·s)	-1.65	0.74	-0.53	0.81	0.005
$\Delta G^{\neq E}$ (J · mol^{-1})	-3477.83	570.69	-9.24	1115.21	12.55
313.15 K					
V^E / (cm^3 · mol^{-1})	2.64	0.79	-1.38	-0.62	0.017
$\Delta\eta$ / (mPa·s)	-1.31	0.74	-0.54	0.33	0.005
$\Delta G^{\neq E}$ (J · mol^{-1})	-3280.39	1052.26	-435.75	435.43	9.67
toluene (1) + 2-methyl-1-propanol (2)					
293.15 K					
V^E / (cm^3 · mol^{-1})	2.20	0.31	0.07	-0.11	0.006
$\Delta\eta$ / (mPa·s)	-4.70	2.79	-1.96	1.46	0.022
$\Delta G^{\neq E}$ (J · mol^{-1})	-3328.55	588.81	-99.24	519.06	21.41
298.15 K					
V^E / (cm^3 · mol^{-1})	2.14	0.36	-0.12	-0.16	0.007
$\Delta\eta$ / (mPa·s)	-4.05	2.75	-1.62	0.94	0.020
$\Delta G^{\neq E}$ (J · mol^{-1})	-3476.11	1667.81	438.95	-435.99	31.20
303.15 K					
V^E / (cm^3 · mol^{-1})	2.09	0.46	-0.26	-0.29	0.008
$\Delta\eta$ / (mPa·s)	-3.32	1.78	-1.27	1.90	0.023
$\Delta G^{\neq E}$ (J · mol^{-1})	-3287.94	533.36	548.75	2157.53	31.15
308.15 K					
V^E / (cm^3 · mol^{-1})	2.02	0.41	-0.35	-0.08	0.005
$\Delta\eta$ / (mPa·s)	-2.81	1.43	-0.90	1.40	0.013
$\Delta G^{\neq E}$ (J · mol^{-1})	-3224.01	181.96	812.06	2584.88	24.13
313.15 K					
V^E / (cm^3 · mol^{-1})	1.97	0.42	-0.48	-0.05	0.007
$\Delta\eta$ / (mPa·s)	-2.37	1.50	-0.67	0.32	0.011
$\Delta G^{\neq E}$ (J · mol^{-1})	-3364.35	1181.76	889.29	195.07	25.73

Table S8. Apparent molar volumes $V_{\phi,1}$ ($cm^{3}\cdot mol^{-1}$) for binary systems at T = 293.15-313.15 K

x_1	T/(K)				
	**293.15**	**298.15**	**303.15**	**308.15**	**313.15**
toluene (1) + 2-propanol (2)					
0.0000	-	-	-	-	-
0.1020	109.60	109.97	110.17	110.78	111.24
0.2001	110.58	111.08	111.57	112.04	112.52
0.3004	111.56	112.06	112.62	113.15	113.68
0.4030	112.50	113.03	113.62	114.19	114.76
0.5051	113.39	113.90	114.55	115.15	115.72
0.6029	114.16	114.69	115.36	115.98	116.57
0.7052	114.94	115.46	116.16	116.80	117.40
0.7998	115.66	116.18	116.91	117.56	118.18
0.9005	116.45	116.97	117.72	118.39	119.01
1.0000	117.23	117.75	118.52	119.20	119.83
toluene (1) + 2-methyl-1-propanol (2)					
0.0000	-	-	-	-	-
0.1000	108.95	109.36	109.85	110.17	110.68
0.1969	109.56	109.99	110.51	110.95	111.48
0.3008	110.2	110.7	111.26	111.78	112.34
0.3922	110.79	111.31	111.92	112.46	113.06
0.4947	111.45	111.99	112.64	113.20	113.82
0.5952	112.12	112.67	113.36	113.92	114.56
0.6936	112.79	113.34	114.05	114.63	115.28
0.7941	113.48	114.04	114.78	115.37	116.04
0.8965	114.21	114.78	115.54	116.14	116.83
1.0000	114.97	115.55	116.34	116.94	117.64

Table S9. Apparent molar volumes $V_{\phi,2}$ ($cm^{3\cdot}$ mol^{-1}) for binary systems at T = 293.15-313.15 K

x_1	T/(K)				
	**293.15**	**298.15**	**303.15**	**308.15**	**313.15**
toluene (1) + 2-propanol (2)					
0.0000	76.46	76.80	77.31	77.75	78.16
0.1020	76.29	76.61	77.09	77.49	77.88
0.2001	76.19	76.51	76.95	77.34	77.71
0.3004	76.16	76.47	76.89	77.27	77.62
0.4030	76.20	76.52	76.91	77.28	77.63
0.5051	76.34	76.63	77.01	77.37	77.69
0.6029	76.48	76.77	77.12	77.47	77.78
0.7052	76.57	76.78	77.13	77.46	77.69
0.7998	76.53	76.76	77.08	77.39	77.64
0.9005	76.61	76.88	77.14	77.40	77.68
1.0000	-	-	-	-	-
toluene (1) + 2-methyl-1-propanol (2)					
0.0000	92.49	92.95	93.59	94.07	94.64
0.1000	92.39	92.83	93.44	93.89	94.44
0.1969	92.32	92.74	93.32	93.76	94.29
0.3008	92.25	92.67	93.23	93.67	94.18
0.3922	92.23	92.63	93.19	93.62	94.12
0.4947	92.21	92.61	93.16	93.58	94.07
0.5952	92.25	92.64	93.17	93.57	94.06
0.6936	92.30	92.66	93.18	93.56	94.00
0.7941	92.33	92.68	93.18	93.57	93.99
0.8965	92.48	92.83	93.27	93.61	94.07
1.0000	-	-	-	-	-

Table S10. Partial molar volumes $\overline{V}_1$ ($cm^{3\cdot}$ mol^{-1}) for binary systems at T = 293.15-313.15 K

	T/(K)				
x_1	**293.15**	**298.15**	**303.15**	**308.15**	**313.15**
toluene (1) + 2-propanol (2)					
0.0000	108.63	108.83	109.22	109.20	109.57
0.1020	108.00	108.34	108.75	108.96	109.41
0.2001	107.58	108.01	108.43	108.79	109.29
0.3004	107.26	107.75	108.20	108.65	109.17
0.4030	107.03	107.55	108.02	108.52	109.06
0.5051	106.85	107.38	107.87	108.40	108.94
0.6029	106.70	107.24	107.75	108.28	108.83
0.7052	106.57	107.10	107.62	108.16	108.71
0.7998	106.46	106.99	107.52	108.05	108.60
0.9005	106.37	106.90	107.44	107.96	108.52
1.0000	106.33	106.87	107.40	107.93	108.49
toluene (1) + 2-methyl-1-propanol (2)					
0.0000	108.40	108.69	109.06	109.27	109.61
0.1000	107.97	108.31	108.70	109.05	109.46
0.1969	107.61	108.00	108.42	108.85	109.31
0.3008	107.28	107.72	108.17	108.65	109.16
0.3922	107.04	107.52	107.99	108.50	109.02
0.4947	106.82	107.32	107.82	108.34	108.88
0.5952	106.64	107.16	107.68	108.20	108.76
0.6936	106.51	107.04	107.57	108.09	108.65
0.7941	106.41	106.95	107.48	108.01	108.57
0.8965	106.36	106.89	107.42	107.95	108.51
1.0000	106.33	106.87	107.40	107.93	108.49

Table S11. Partial molar volumes $\overline{V}_2$ ($cm^{3\cdot}$ mol^{-1}) for binary systems at T = 293.15-313.15 K

x_1	T/(K)				
	**293.15**	**298.15**	**303.15**	**308.15**	**313.15**
toluene (1) + 2-propanol (2)					
0.0000	76.46	76.80	77.31	77.75	78.16
0.1020	76.50	76.85	77.35	77.79	78.21
0.2001	76.63	76.97	77.47	77.92	78.32
0.3004	76.84	77.18	77.67	78.12	78.51
0.4030	77.13	77.46	77.94	78.39	78.76
0.5051	77.47	77.78	78.26	78.69	79.03
0.6029	77.82	78.10	78.57	78.97	79.30
0.7052	78.15	78.40	78.85	79.23	79.53
0.7998	78.38	78.60	79.03	79.40	79.68
0.9005	78.48	78.68	79.07	79.44	79.73
1.0000	78.36	78.54	78.89	79.28	79.59
toluene (1) + 2-methyl-1-propanol (2)					
0.0000	92.49	92.95	93.59	94.07	94.64
0.1000	92.51	92.98	93.61	94.10	94.67
0.1969	92.58	93.05	93.69	94.18	94.74
0.3008	92.71	93.18	93.82	94.31	94.87
0.3922	92.87	93.34	93.99	94.46	95.02
0.4947	93.10	93.57	94.21	94.67	95.23
0.5952	93.38	93.83	94.47	94.91	95.45
0.6936	93.70	94.12	94.74	95.17	95.70
0.7941	94.07	94.45	95.04	95.46	95.96
0.8965	94.50	94.81	95.32	95.76	96.23
1.0000	94.96	95.17	95.59	96.07	96.50

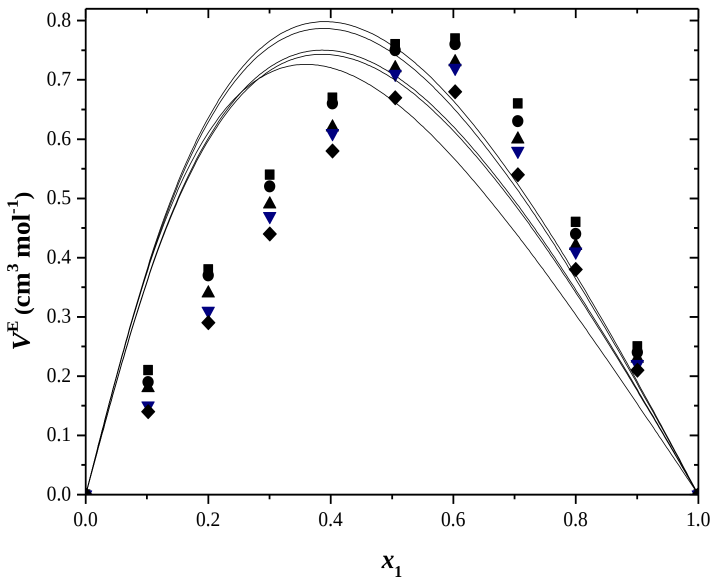

Figure S7. Excess molar volumes (V^E) versus mole fraction for toluene (1) + 2-propanol (2) system at: ■ 293.15 K; ● 298.15 K; ▲ 303.15 K; ▼ 308.15 K; ♦ 313.15K. The solid curve was calculated from PFP theory.

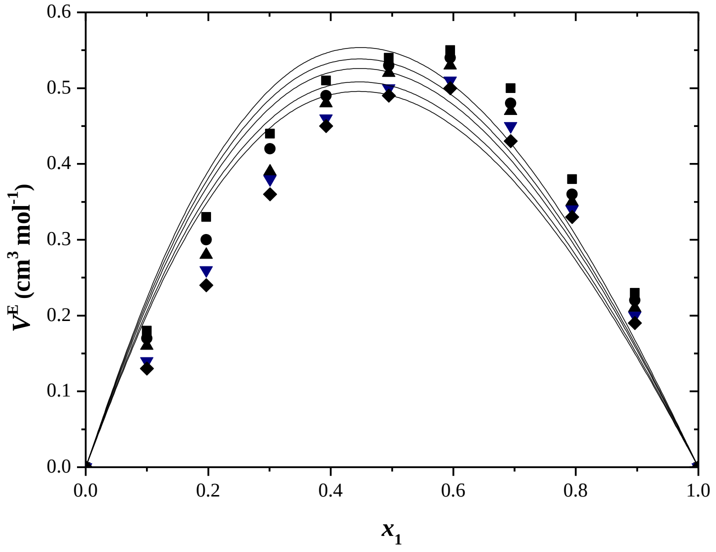

Figure S8. Excess molar volumes (V^E) versus mole fraction for toluene (1) + 2-propanol (2) system at: ■ 293.15 K; ● 298.15 K; ▲ 303.15 K; ▼ 308.15 K; ♦ 313.15K. The solid curve was calculated from PFP theory.
